# Unconscious Local Motion Alters Global Image Speed

**DOI:** 10.1371/journal.pone.0112804

**Published:** 2014-12-15

**Authors:** Sieu K. Khuu, Charles Y. L. Chung, Stephanie Lord, Joel Pearson

**Affiliations:** 1 The School of Optometry and Vision Science, The University of New South Wales, Kensington, New South Wales, Australia, 2052; 2 The School of Psychology, The University of New South Wales, Kensington, New South Wales, Australia, 2052; University of Tokyo, Japan

## Abstract

Accurate motion perception of self and object speed is crucial for successful interaction in the world. The context in which we make such speed judgments has a profound effect on their accuracy. Misperceptions of motion speed caused by the context can have drastic consequences in real world situations, but they also reveal much about the underlying mechanisms of motion perception. Here we show that motion signals suppressed from awareness can warp simultaneous conscious speed perception. In Experiment 1, we measured global speed discrimination thresholds using an annulus of 8 local Gabor elements. We show that physically removing local elements from the array attenuated global speed discrimination. However, removing awareness of the local elements only had a small effect on speed discrimination. That is, unconscious local motion elements contributed to global conscious speed perception. In Experiment 2 we measured the global speed of the moving Gabor patterns, when half the elements moved at different speeds. We show that global speed averaging occurred regardless of whether local elements were removed from awareness, such that the speed of invisible elements continued to be averaged together with the visible elements to determine the global speed. These data suggest that contextual motion signals outside of awareness can both boost and affect our experience of motion speed, and suggest that such pooling of motion signals occurs before the conscious extraction of the surround motion speed.

## Introduction

An important task of the visual system is to derive an estimate of the motion (speed and direction) of objects in the three-dimensional (3D) visual scene. Research over the last 30 years has established that the detection and analysis of motion occurs in at least two computational steps [1]. Initially, local estimates of motion are derived, and then at a later stage, these estimates are integrated to form global motion [2]. It is believed that this two-stage analysis of motion is processed along the dorsal visual pathway projecting from primary visual cortex (V1). Cells in V1, which are restricted in the spatial extent of their analysis, initially obtain an estimate of local motion, before outputting to higher cortical areas located along the dorsal pathway, such as middle temporal lobe (MT) [2] and medial superior temporal lobe(MST), [3]. Cells in these areas have large receptive fields, and are thought to function by integrating local motion estimates to derive an estimate of the global or overall direction and speed of objects.

The role of conscious awareness in motion processing has recently garnered much attention [4], [5]. Under appropriate conditions, visual information rendered ‘invisible’ continues to be processed by the visual system [6], [7], [8], [9]. For example, behavioural studies using binocular rivalry have demonstrated that binocular suppression of an adapting stimulus does not eliminate its ability to generate a motion after-effect (MAE) in a subsequent test stimulus [10], [11], [12]. This demonstrates that the processing of motion information can occur without visual awareness. These studies raise both computational and philosophical issues about the role of consciousness in visual perception. There is a need to investigate non-conscious vision to reveal how it may contribute to the conscious perception of a scene.

While studies have shown that, under appropriate conditions, motion processing can occur without visual awareness, the extent of this effect is dependent on a number of stimulus factors. In particular, motion adaptation during binocular rivalry suppression is dependent on stimulus properties such as the contrast of [13], and the type of motion [14] in the adapting stimulus. With regard to the latter, motion after-effects are most demonstrable when the adapting motion is unidirectional and simple. Weisenfelder & Blake [14] reported that adaptation to spiral motion occurred only when the adapting stimulus was dominant and visible to the observer. When the adapting stimulus was suppressed from view, it did not contribute to the adaptation process. It is believed that complex motion is processed in higher cortical stages and reflects global pooling of local motion estimates [15], [16], [17]. As the detection of complex motion requires the visual system to spatially integrate local motion, Blake [18] concluded that rivalry suppression might affect the binding/integration of local features to detect global complex motion. This raises the possibility that detection and processing of local motion might function without the need for conscious perception, while the higher stages of motion analysis that operate by integrating local signals might depend on visual awareness.

Note that the failure to generate an MAE from adaptation to complex motion does not rule out the possibility that local motion information is integrated under binocular suppression. It is possible that motion integration under binocular suppression is attenuated such that it is insufficient to generate a compelling MAE, which in itself is a weak motion percept [19], [20]. An attenuated motion integration process will produce a weaker adapting stimulus that might not be sufficient to activate and adapt high-level neurons tuned to complex motion. This is further complicated by the fact that binocular rivalry is bistable: the short phases in which the adapting stimulus is suppressed might be insufficient to increase the MAE. Kaunitz et al. [21] addressed this limitation by measuring the MAE produced by adaptation to a complex (spiral) motion stimulus made invisible using the more powerful method of Continuous Flash Suppression (CFS) [7] to achieve prolonged stable visual suppression. In this method, a flickering mask (e.g., a Mondrian pattern changing at approximately 10 Hz) is presented to one eye, and the to-be-suppressed target image to the other. Under CFS, the observer perceives the flickering mask, not the adapting stimulus. Kaunitz et al. [21] demonstrated that despite CFS suppression, adaptation to complex motion still produced an MAE, although it was attenuated relative to visible conditions. Unconscious motion processing has been further corroborated by recent findings that highly coherent global motion patterns break CFS more often than patterns with no global motion coherence [22], [23]. This suggests that the processing of complex motion might occur implicitly without the need for visual awareness. However the stimulus conditions under which it arises requires further exploration.

While the observation has been made that global motion can be processed without awareness, the consequences and implications of this remain largely unknown. In particular, how unconscious motion might contribute to and affect conscious visual perception remains poorly understood. While conscious visual perception might be an active process modulated by attention, it has been well demonstrated that unconscious information can influence a number of explicit behavioural judgements [24], [25], [26], [27], [28], [29], [30]. In the present study we investigated how unconscious motion signals affect concurrent global conscious motion perception. Note that much of the research on motion processing without awareness has utilised MAEs. While measuring MAEs is indeed an effective method for probing the activation of motion mechanisms under visual suppression, it does not directly reveal how unconscious motion might affect *immediate* conscious motion perception. Given that for observers to successfully interact with the visual world, the visual system must expediently derive an estimate of motion, it is important to establish the degree to which unconscious motion information contributes to this process. In the present study we sought to address this issue and examined the ability of the visual system to derive an estimate of an object's global speed when the local motion elements were suppressed from awareness.

Image speed directly informs about the rate of change in the spatial position of an object moving to a given location [31], [32], [33]. This information is most useful in predictive behavioural judgements concerning the time of arrival/contact of/with objects. Most critical to the aims of the present study, previous studies have demonstrated that the perception of object speed is derived through the integration of local estimates. For example, Verghese and Stone [34] showed that the ability of the visual system to discriminate the speed of a multi-element stimulus (a cluster of Gabors) depends on the number of local elements that form the object; discrimination thresholds decreased monotonically as the number of local elements increased. This finding was attributed to local elements being treated as independent speed samples, with spatial integration reducing uncertainty and improving reliability when estimating the global speed. Similarly, spatial integration of local motion is reflected in the judgment of apparent/perceived speed [31] [35] [33]. For example, using a version of the global dot motion stimuli (GDM) Khuu and Badcock [31] demonstrated that the perceived global speed of a complex pattern (e.g., rotational and radial motion) is consistent with the average of two different speed populations. This finding provides further corroborating evidence that local motion signals are initially extracted before integration, and averaged at a higher stage of processing to determine the global speed of motion.

**Figure 1:**
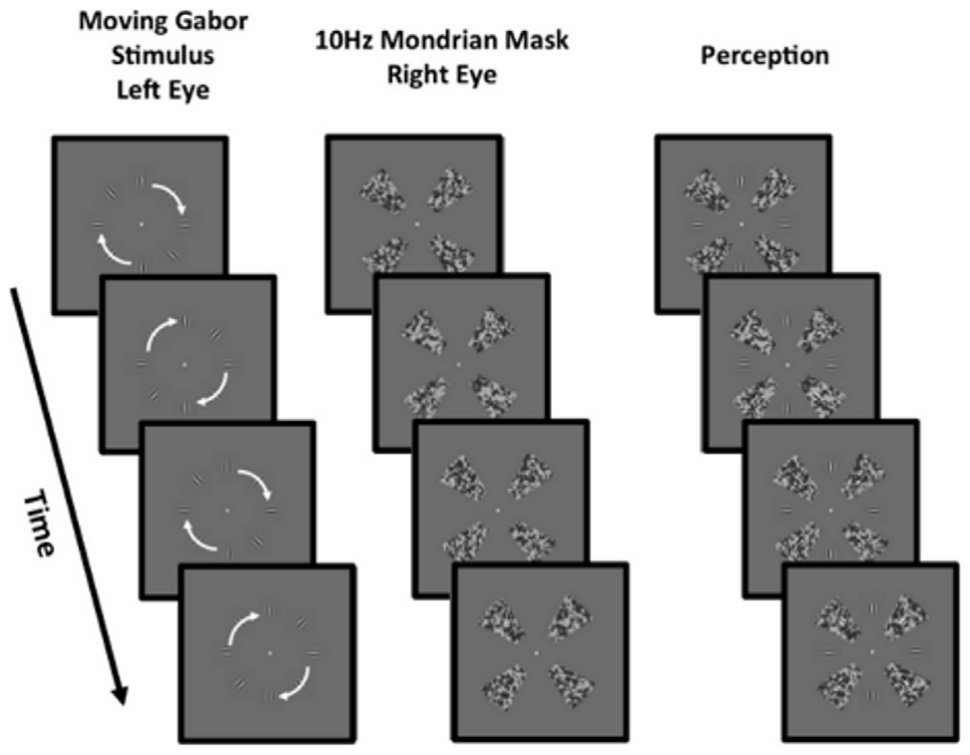
A schematic of the stimulus used in the present study. The moving Gabor stimuli undergoing rotational local motion was presented to the left eye while a dynamic Mondrian mask was presented to the right eye. When viewed binocularly perception was of a Gabor stimulus with half of its elements masked by the Mondrian stimulus.

In the present study we quantified the perception of image speed, as this is useful in providing a *direct* means of assessing the immediate integration of local motion. Following the approaches of Verghese and Stone [28], and Khuu and Badcock [31], the present study examined the discrimination and judgment of global speed, and determined whether visual awareness of local estimates is necessary for their integration. Thus, we question the immediate availability of unconscious signals to visual perception and the degree to which these signals might influence the judgement of visible motion. Observers were presented with an annulus of Gabors elements (see Fig. 1), and CFS was used to systematically vary the number of local elements that were suppressed from awareness. Note that the stimulus always contained visible local motion elements, and we questioned whether the motion of invisible elements contributes to their perception. In Experiment 1 we determined whether this ‘invisible’ motion affects the ability to discriminate the speed of a visible moving stimulus. In Experiment 2 we examined whether global speed averaging persists under conditions in which a subset of Gabors were suppressed from awareness. If suppressed local motion were available and contributes to spatial integration, masking local elements from awareness ought not to affect the ability of the visual system to discriminate and judge the apparent speed of a global motion stimulus. Alternatively, if awareness of local elements is required for their integration, discrimination thresholds and perceived speed judgements ought to change with the number of suppressed elements. Significantly, the method of the present study represents a departure from previous paradigms that have quantified the MAE *after* a period of adaptation to a motion stimulus that is *completely* suppressed from visual awareness. The advantage of our method is that it assesses the role of visual awareness in the immediate integration of motion (N.B., the MAE is an after product of motion integration) for the perception of global speed, as well as the degree to which unconscious local signals might contribute to this perception.

### Ethics Statement

All participants in this and the following experiments gave written consent in advance, to procedures approved by The University of New South Wales Human Research Ethics Committee, outlined in the Australian National Statement on Ethical Conduct in Human Research (2007).

## Experiment 1: Global Speed Discrimination without Visual Awareness

Verghese and Stone [34] reported that increasing the number of local elements in the stimulus improved global speed discrimination. Greater motion sensitivity arises because the integration of many elements reduces uncertainty and minimises noise. Using the discrimination of global speed as a measure of motion integration, Experiment 1 determined whether the systematic suppression of local elements modulates global speed discrimination thresholds. These results were compared to a baseline condition in which global discrimination thresholds were measured using a stimulus in which the physical number of Gabors was modulated. If visual awareness is essential for the discrimination of global speed, discrimination thresholds ought to increase with the number of locally suppressed elements in a manner consistent with a physical change in the number of elements in the stimulus. That is, local elements do not contribute to the estimation of global motion when they are suppressed from awareness. However, if invisible motion contributes to perception, thresholds ought to be analogous to when all elements are visible, and remain little changed with the number of suppressed elements.

### Methods

#### Observers

Six observers with normal or corrected-to-normal visual acuity participated. All were experienced observers, but naive to the purpose of the present study.

#### Stimuli

The stimulus (see Fig. 1 which shows the condition in which 4 elements were visible and 4 were masked using CFS) was an 8-frame movie sequence displaying an annulus of stationary ‘local motion’ Gabor elements that were placed on a mid grey background of (40 cd/m^2^) and spaced equally (at 45° intervals) on the circumference of a circle with a radius of 4°. The position of these elements was not fixed across trials, but randomised by shifting all elements by a fixed angular value. This ensured that observers could not anticipate where stimulus elements might appear from trial to trial. Each Gabor was given by the product of a circular Gaussian and an oriented sinusoid: G(x, y)  =  e –(x^2^ + y^2^)/2s^2^ * cos[2p * (cos q * x + sin q * y)/p + f] where *q* is the orientation and *f* determines the relative phase of the element. The size of the element, which is defined by *s*, was approximately 0.75° of visual angle at full width and at half-height, and the spatial frequency or period (*p*) of the modulating sinusoid was set to 1 cycle per degree. The Michelson's contrast of the Gabor elements was 0.74. To generate motion, the phase of the sinusoid was systematically changed (at different rates to produce different speeds) across 8 movie frames (see below), with each frame shown for 33 ms with no inter-stimulus interval. Thus the total duration of the stimulus was 264 ms. Additionally to investigate differences in the type of motion pattern, the local orientation of the Gabor elements was changed in different conditions to simulate translational motion (all Gabors were vertically aligned) or rotational motion (Gabors aligned along radii from the centre of the stimulus).

The masking of elements was achieved using the CFS technique devised by Tsuchiya & Koch [7]. The Gabor stimulus was presented to the non-dominant eye (as measured using the Miles test for sighting dominance) while the other eye was presented with a dynamic black and white Mondrian mask (see Fig. 1) that flickered at 10 Hz — the same temporal frequency as the moving stimulus. The mask segments were ‘windmill’ in shape (subtending an angle of 30° with a length of 2.5°) and consisted of numerous overlapping grey squares (Weber contrast: between −0.74 and 0.74) that were positioned randomly in the mask. The mask segments were placed at a distance of 4° from the centre of the stimulus and spatially positioned so that they completely overlapped with the Gabor elements presented to the other eye. This method allowed us to *selectively* suppress a select proportion of the elements from awareness. This process of selectively suppressing components of the stimulus from awareness using CFS has been adopted in previous studies that have investigated whether the effects of visual crowding and the formation of illusory contours (in Kanizsa figures) depends on the visual awareness of elements that induce these effects [36] [37]. To aid binocular fusion and minimize eye vergence, the stimulus was bordered by a black (10 cd/m^2^) rectangular frame (line width: 0. 5°, line length 8°) positioned at 0 disparity. Stimuli were generated using custom software in MATLAB (version 9), and the stimulus was presented centrally on the screen of a linearised 3D monitor whose background colour was grey and set to a luminance of 40 cd/m^2^. Observers viewed the stimulus at a distance of 60 cm through polarized lenses.

### Procedures

**Figure 2:**
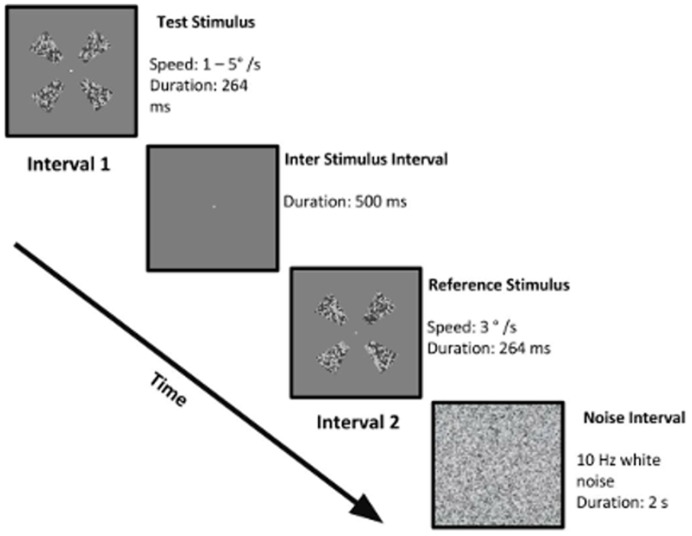
A schematic diagram of the two interval stimulus presentation sequence. In one interval was the test stimulus which consisted of Gabor elements all moving at a speed between 1–5°/s. In the other interval a reference stimulus was presented that consisted of Gabor elements moving at 3°/s. Both intervals were separated by an inter stimulus interval of 500 ms in which the screen was mid gray set to the background luminance. The task of the observer was to indicate the interval containing the fastest motion. After the presentation of both intervals, dynamic white noise was presented.

In Experiment 1, a two-interval forced-choice (2IFC) procedure was used in conjunction with Method of Constant Stimuli (MoCs) to quantify the effect of changing the number of visible elements (either through suppression or by actually changing the number of elements) on speed discrimination thresholds. Observers were presented with pairs of the Gabor stimuli moving at different speeds in separate temporal intervals (see Fig. 2 for a schematic diagram of the stimulus presentation sequence). In one interval, a test Gabor stimulus moved at one of 9 speeds: 1, 1.5, 2, 2.5, 3, 3.5, 4, 4.5, and 5°/s. In the other interval, a reference Gabor stimulus was presented in which all Gabors moved at a constant speed of 3°/s. The presentation order of the stimulus intervals was randomised between trials, and separated by brief period of 500 ms in which the screen was blank and displayed the background luminance.

The task of the observer was to indicate the interval containing the fastest moving Gabors. Each test stimulus speed was repeated 50 times. Though the stimuli were presented briefly, each trial was succeeded by a square containing dynamic white noise (9°x 9°), presented for 2 seconds binocularly, to prevent afterimage formation (see Fig. 2). This procedure was repeated in two experimental conditions. In the first condition, in a replication of Verghese and Stone [34], speed discrimination thresholds were measured separately for stimuli consisting of 2, 4, 6 and 8 Gabor-elements. Gabor elements were equally spaced on the circumference of the circle with pairs of Gabors located at polar opposites. For this condition, it was expected that speed discrimination would improve as the number of elements increased. This condition provides a direct measure of how local elements are spatially integrated in the discrimination of speed, and serves as a baseline for comparison conditions in which local elements were instead masked. The second condition was similar to the first, but the number of visible elements comprising the stimulus was always 8 and the visibility of elements was changed using CFS. Rather than physically removing/adding elements, they were instead binocularly suppressed by changing the number of Mondrian patches so that 0, 2, 4 and 6 elements were invisible per trial. Note that in all conditions visible elements were present in the stimulus and on which observers attended to. If suppression was broken in a particular trial, such that moving Gabors became clearly visible (which the observer indicated by pressing the keyboard), it was presented again at a random interval within the MoCs sequence. Note that moving Gabors rarely broke CFS suppression, which occurred on average less than 2% of trials. The observation that good suppression (such that observers only saw of the Mondrian mask) occurred in the majority of trials might be because the stimulus duration was very brief at 264 ms, and in addition, Gabors were placed at peripheral and discrete locations (4 degrees) where spatial vision might be sufficiently coarse to reduce stimulus visibility. Indeed, the duration of the stimulus used in the present study was much smaller than that reported to break CFS suppression (2–4 seconds), by systematically increasing the contrast of the stimulus [21] [22].

Blake [18] noted that translational motion is effective in activating local detectors, while rotational motion requires spatial pooling for its detection and therefore engages neural mechanisms in higher cortical areas [16]. Accordingly, a difference might be observed in the effect of CFS on the perception of these motion types. To examine this possibility, we repeated the aforementioned procedures and included translational and rotational complex motion as independent variables. In total there were 16 MoCs trials comprising 4 different Gabor-element numbers, which was repeated for the two experimental conditions and for the two motion types. These trials were performed in a randomized order with breaks in between trials to avoid fatigue.

### Results and Discussion

**Figure 3:**
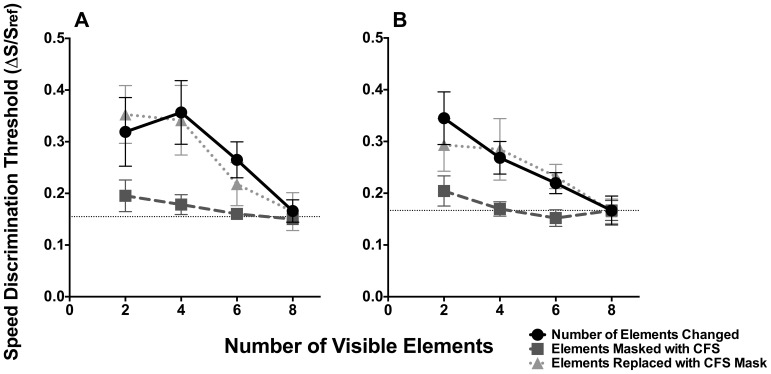
Speed discrimination thresholds for translational motion (A) and rotational motion (B). Speed discrimination thresholds as Weber fractions plotted as a function of the number of elements visible in the stimulus. Circles depict data for conditions in which the actual number of elements in the stimulus was changed; diamonds - removed elements were replaced by a dynamic Mondrian patch; and circles elements are present but suppressed from awareness using CFS. Error bars represent 95% confidence intervals.

The proportion of times that the test stimulus was judged to be moving faster than the reference was determined for the 9 different speeds. The psychometric function was estimated by logistic fits to data using GraphPad Prism (version 6). The speed discrimination threshold was defined as half the speed difference required for performance to change from the 25% to 75% level on the psychometric function. This analysis was repeated for the different Gabor element numbers, and for experimental conditions in which the number of elements was suppressed or removed from the stimulus. Speed discrimination thresholds, expressed as a Weber fraction (ΔS/S_ref_, where ΔS is the speed discrimination threshold, and S_ref_ is the speed of the reference stimulus: 3°/s), are plotted in Fig. 3 as a function of the number of visible elements for translational (Fig. 3A) and rotational motion (Fig. 3B). The data trend between observers was similar, so the averaged data are shown (error bars signify 95% confidence intervals). The results for experimental conditions in which elements were suppressed from awareness (CFS condition) or physically changed (baseline condition) are shown in Fig. 3 as squares and circles respectively.

A repeated measures two-way ANOVA was performed to determine whether changing the number of visible elements (factor 1: 0, 2, 4, and 6) and whether elements were masked or removed from the stimulus, significantly affected speed discrimination thresholds. This analysis was conducted separately for translational and rotational motion. For translational motion, a main effect was observed for the number of visible Gabor elements (F(3, 45) = 34.06, p<0.0001) and between conditions in which local elements were changed or suppressed from awareness using CFS (F(2, 15) = 31.11, p<0.0001; Fig. 2 panel A) . Likewise with rotational motion: main effects were observed when the number of visible Gabor elements was systematically changed (F(3, 45) = 36.53, p<0.0001) and when the method with which this was achieved was altered (CFS vs changing the number of elements, F(2,15) = 35.30, p<0.0001; Fig. 2 panel B). In addition a significant interaction effect was also observed for both translational (F(6,45) = 3.04, p = 0.0139) and rotational motion (F(6,45) = 3.58, p = 0.0055). Thus for both motion types, the effect of changing the number of elements on speed discrimination thresholds was dependent on the method used to change their visibility. As noted, speed discrimination thresholds for the CFS condition were much lower than for conditions in which the actual number of Gabors comprising the stimulus was changed. Indeed Tukey's post-hoc tests corrected for multiple comparisons showed that the mean threshold between the two conditions (CFS vs baseline conditions) was significantly different (ps<0.01) for stimuli consisting of 2, 4 and 6, but not at 8 elements. This outcome was similar for both motion types. Note that both experimental conditions were the same when they consisted of 8 elements, and consequently accounts for the convergence of both functions to this point.

These data indicate that the ability to discriminate global speed is contingent on the number of physical elements, largely independent of awareness. For both translational and rotational motion, when the number of Gabor elements in the stimulus was increased (circles) there was a noticeable improvement (i.e., a reduction) in speed discrimination thresholds. This replicates Verghese and Stone [34], and demonstrates that the visual system spatially integrates local speed information to facilitate the discrimination of global object speed. As noted by Verghese and Stone [34], this improvement in performance reflects the fact that each local element is treated as an independent sample of the object's speed; their integration reduces the noise in estimating motion quantity. However, when Gabor elements were rendered invisible using CFS, a different data trend was observed: speed discrimination thresholds were immediately superior under CFS conditions. This was the case for both translational and rotational motion. For CFS conditions, speed discrimination thresholds for stimuli in which 2, 4 and 6 elements were approximately 2 times lower than for conditions containing the same number of physical elements. This indicates that the visual system was better able to discriminate the speed of stimuli in which local elements were suppressed from awareness than those in which the actual number of elements comprising the stimulus was changed. This finding indicates that despite local motion elements being suppressed from visual awareness, they continue to be integrated (along with visible elements) by the visual system to derive an estimate of global speed.

In CFS conditions increasing the number of visible elements also improved speed discrimination performance (one-way ANOVA (with Geisser-Greenhouse correction): translational motion (F(3,20) = 4.44, p = 0.0370: rotational motion: F(3,20) = 7.98, p = 0.0081), though not at the same rate as when they were physically changed. This finding suggests that while suppressed elements are integrated to affect speed discrimination thresholds, their contribution is not entirely optimal. Note that optimal integration would mean that thresholds would not change with the number of elements and be similar to when all 8 elements are visible. However, for both motion types, Tukey's post-hoc comparisons (corrected for multiple comparisons at an alpha of 0.05) revealed that while there was a significant difference between 2 and 8 element conditions (ps<0.036), other comparisons between different element conditions were not significant (ps>0.1634). This finding suggests that the attenuation of motion integration is small and most evident when only a few elements in the stimulus are visible. The finding that binocularly suppressing elements from awareness attenuates the degree to which they contribute to motion is consistent with the findings of Kaunitz et al. [21] who showed that CFS suppression produces a weaker MAE as compared to when the adapting stimulus was visible.

While the finding that speed discrimination thresholds under CFS conditions improved with increasing the number of elements is consistent with a partial/sub-optimal integration process, there is an alternative explanation. In the CFS conditions, a dynamic Mondrian mask was used to selectively mask local Gabor elements. While this mask is effective in suppressing local elements, the dynamic texture of the mask (which is visible to the observer) might unintentionally affect performance by contributing motion noise. Thus, elevated thresholds might be because the visual system integrates the motion noise of the Mondrian mask in addition to invisible Gabor elements. Thus, ‘partial’ summation observed in CFS conditions might be attributed to the influence of the mask, and be dependent on the number of Mondrian patches comprising the mask. To test this possibility we conducted a supplementary experiment in which we determined the degree to which the motion noise of the Mondrian patches might affect the ability to discriminate global object speed.

In this supplementary experiment we repeated the baseline condition, but the removed Gabor elements were physically replaced with Mondrian patches. Perceptually, the stimulus used in this supplementary experiment was identical to the original CFS stimulus, but no Gabor elements were presented to the other eye, (i.e., there were no invisible elements; speed discrimination thresholds were derived exclusively from unmasked visible elements). If the dynamic mask contributed noise to the speed discrimination process, thresholds for this condition would be much higher than for the original baseline condition, which did not contain any Mondrian patches (circles).

The same six observers performed the supplementary experiment, their results are shown in Fig. 3A and 3B (grey triangle data points dashed line, error bars signify 95% confidence intervals). For both translational and rotational motion, speed discrimination thresholds were no different from the baseline conditions (two-way ANOVA, translational motion: F(3,30) = 0.25, p = 0.864; rotational motion: F(3,30) = 0.23, p = 0.874). This result indicates that the ability to integrate the speed of local elements to discriminate the global speed was unlikely to be affected by the motion noise of the Mondrian mask. Thus the visual system is able to perceptually segregate the *coherent* motion of Gabor elements from the motion noise of the mask, and treat them as independent samples. These results show that the partial summation observed in the CFS condition in the main experiment is due to attenuation of the integration of elements under binocular suppression, and not the motion noise from the mask itself.

To ensure that our CFS mask achieved maximum suppression of the moving Gabors we conducted a control experiment to quantify the effectiveness of the CFS mask. Note that while our stimulus rarely broke suppression completely (as noted previously), it cannot be directly verified what observers perceived when the stimulus was masked by CFS. Indeed, it might be possible that the motion of the Gabors remains perceptible because it is partially visible, or, critically, it is combined (through binocular summation) with the dynamic noise in the CFS mask. In the latter condition the dynamic noise of the CFS mask coinciding with the location of the suppressed targets might adopt characteristics of the Gabor motion (akin to motion capture). Under these circumstances the local Gabor motion might be integrated by the visual system. To examine the effectiveness of our CFS mask, the previously described procedures were again used with local elements of both the reference and test stimuli completely masked by CFS. Under these conditions observers (the same six observers who participated in the main experiment) were required to make a speed judgement regarding the interval containing the fastest moving stimulus. If the motion of Gabors was visible, observers would should be able to discriminate its apparent speed. However, if the CFS mask effectively made local elements invisible then the expectation is that observers will perform at chance. This procedure was repeated 50 times for stimuli comprised of 2, 4, 6 and 8 elements in a randomised order. As in the main experiment if the stimulus completely broke suppression (this scenario was not of interest in the control experiment), observers repeated that trial at a random point in the MoCs sequence.

**Figure 4:**
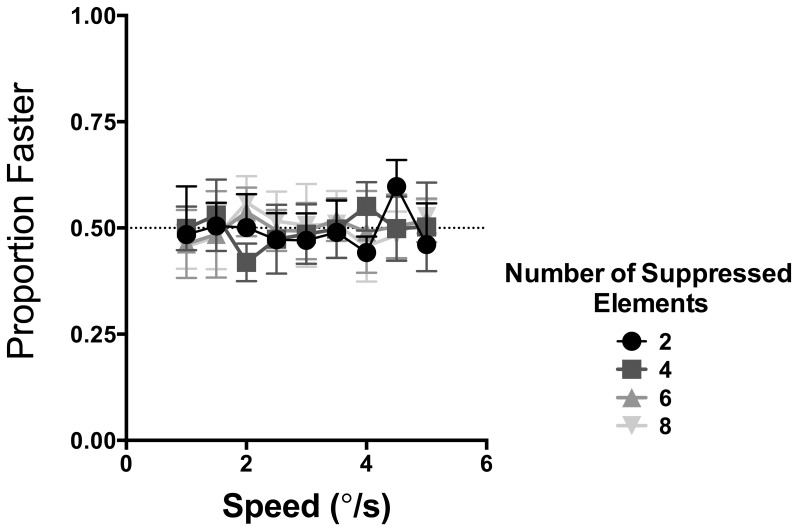
The ability to judge the speed of suppressed elements. The proportion of times in which observers were able to correctly identify the faster moving stimulus comprising of a number of different elements (different symbols) suppressed from awareness plotted as a function of their speed. Error bars signify 95% confidence intervals.

The results of this control condition are shown in Fig. 4. The proportion of times in which observers correctly identified the faster moving stimulus is plotted as a function of the test stimulus speed. The average data are shown with error bars signifying 95% confidence intervals. Different symbols indicate conditions in which the stimulus consisted of 2, 4, 6 and 8 elements. These data show that observers were unable to *consciously* perceive and judge the speed of the masked Gabor elements, with performance at chance level regardless of the number of elements. A two-way repeated measures ANOVA indicated that both factors – the Gabor speed (F(8,45) = 1.244, p = 0.297) and the number of Gabor elements (F(3,135) = 0.0191, p = 0.996) – were not significant and did not affect the judgement of speed. These data also showed that the dynamic Mondrian mask employed in the present study was effective in achieving strong and complete binocular suppression, and that the motion of Gabor elements were not added to the Mondrian motion. We are therefore confident that observers were unlikely to perceive and consciously report on the motion of local Gabors under CFS.

In summary, the results of Experiment 1 suggest that the visual system is able to concurrently integrate visible and invisible local motion to perceive both translational and rotational motion. In CFS conditions the stimulus always comprised 8 elements, and despite the fact elements were suppressed from awareness, they continued to be integrated (with visible elements) by the visual system to influence the perception of global speed. Note, however, that this integration process is not entirely optimal; thresholds do show improvement with the number of visible elements suggesting that there is benefit when elements are unsuppressed and visible to the visual system. These data reflect the possibility that binocular suppression *attenuates,* but does not prevent, the integration of local information for both translational and complex motion forms.

## Experiment 2: Global Speed Averaging without Visual Awareness

Khuu and Badcock [31] observed that, for a complex motion stimulus consisting of local elements moving at different speeds, the visual system averages local estimates provided that the difference in speed is small. Larger speed differences segment the stimulus preventing them from being averaged. In this experiment we examined whether CFS disrupts the ability of the visual system to integrate and average local speed information. We used the same Gabor stimulus as in Experiment 1, but moved local Gabor elements at different speeds. We examine whether unconscious suppressed motion signals modulate conscious motion signals, when unconscious and conscious motion are at different speeds. If motion integration occurs without awareness, the perceived speed of the test stimulus will be consistent with the average of the two different Gabor speeds, despite one group of elements being invisible. However, if suppression prevents the integration of local estimates, global averaging will not be observed, rather the perceived global speed of the stimulus will be derived exclusively from visible Gabor elements.

### Methods

Experiment 2 used the same observers, stimulus and methods as in Experiment 1, but the number of elements forming the stimulus was kept constant at 8. Local Gabor elements were configured to convey complex rotational motion only, and the speed of individual elements was systematically varied such that spatially alternating elements moved at different speeds. Thus, the stimulus comprised of two equal groups of elements that differed in speed. As in Experiment 1, observers were required to judge the speed between two stimuli presented in two separate temporal intervals (as in Fig. 2).

There were three experimental conditions. In the first condition, the test stimulus comprised of 8 Gabor elements all moving at one of 11 speeds: 1, 1.5, 2, 2.5, 3, 3.5, 4, 4.5, 5, 5.5 and 6°/s, presented in a MoCS design. The reference stimulus also comprised 8 elements, but all moved at a constant speed of 3°/s. This condition provided a measure of baseline performance. In the second condition, the test stimulus consisted of local Gabor elements in which alternating elements moved at different speeds: odd numbered Gabors moving at a fixed speed of 2°/s, even numbered Gabors moved over the range of 11 speeds as in Condition 1. As Khuu & Badcock [31] demonstrated, because odd numbered elements are moving at a constant speed that is lower than the reference stimulus speed of 3°/s, even numbered elements are required to move *faster* to match the apparent speed of the reference stimulus. A 4°/s increase in speed is required to be consistent with global averaging. The third condition was the same as the second condition, but odd numbered elements (i.e., those moving at a constant speed of 2°/s) were made invisible using CFS. Note that for this condition, even numbered elements were visible, and (as in the previous conditions) could move over a range of speeds between 1 to 6°/s. The reference stimulus was also similarly suppressed to ensure that both test and reference stimuli perceptually similar to the CFS conditions used in Experiment 1. Here the stimulus comprised of 8 elements, but 4 were suppressed from awareness. Of note is that for this stimulus configuration visible and invisible elements equally contribute to the perception of motion (i.e., there is no attenuation of the integration of motion of invisible elements) as the speed discrimination thresholds between 4 and 8 visible element conditions were the same (see Experiment 1 and Fig. 3).

### Results and Discussion

**Figure 5:**
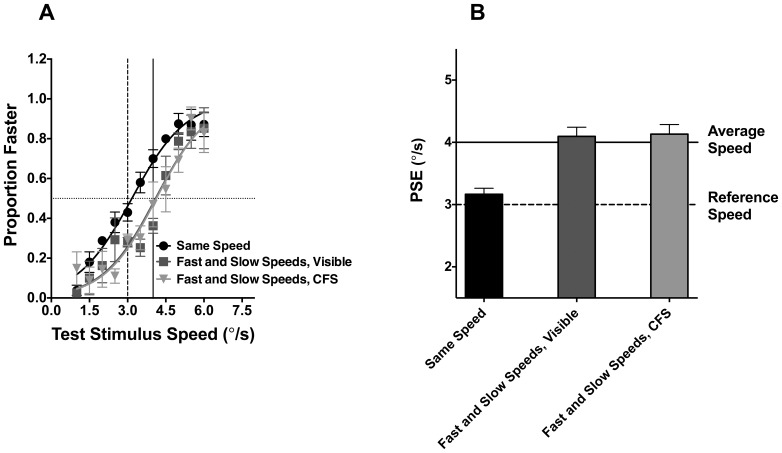
Perceived speed judgments of patterns with visible and invisible elements. The proportion of times in which the test stimulus was judged faster than the reference stimulus is plotted in against the speed of the test stimulus in Fig. 4A; Average observer data is plotted for conditions in which Gabor elements moved at the same speed (circles), when half the elements moved at a slower speed and were visible (triangles) or invisible suppressed by CFS (squares). In Fig. 4B, the PSE for the function fits in Fig. 4A are plotted as bar graphs. In each plot error bars represent 95% confidence intervals.

The proportion of times the test stimulus was judged to be moving faster than the reference stimulus was collated for the different speeds of the test stimulus in the three different conditions. As in Experiment 1, Logistic fits of these data (average R^2^: 0.92) provided an estimate of Point of Subjective Equivalence (PSE) which corresponded to the speed of the test stimulus required to perceptually match the reference stimulus speed (which is indicated in the figure by the solid vertical line). These function fits are shown in Fig. 5A for conditions in which all Gabors in the test stimulus moved at the same speed (circles), half the number of Gabors moved at a slower speed of 2°/s and were visible (squares), or invisible (triangles). Data are the average of the 5 observers and error bars signify 95% confidence intervals. In Fig. 5B, the average observer PSEs for the three stimulus conditions are shown as bar graphs. A one-way ANOVA performed on the average PSE values indicated a significant difference between the three conditions (F(2,14) = 13.62, p = 0.0002). Tukey's Post-hoc pairwise comparison tests (corrected for multiple comparisons) revealed a significant difference between the baseline condition (in which all Gabors moved at the same speed) and the two other mixed speed conditions (ps<0.0001). However, there was no significant difference (p = 0.875) between the PSEs for the two mixed speed conditions. This suggests that the global speed of the stimulus in the two mixed speed conditions were approximately equal, even though for CFS conditions only half the number of the Gabor elements was visible to observers.

The results of Experiment 2 point to a number of findings. First, when Gabors all moved at the same speed (circles), observers accurately judged the speed of the test stimulus such that the PSE was the same as the reference stimulus speed; note that, in Fig. 5B, the PSE for this condition approximates the reference speed (dashed vertical line in Fig. 5A and dashed horizontal line in Fig. 5B). Second, contrasting with the results of the first condition, when the stimulus comprised two different populations of Gabor speeds, the test stimulus had to physically move faster to match the speed of reference stimulus. For this condition the PSE was approximately 4°/s, which is consistent with the visual system integrating and averaging the local speeds (solid vertical line in Fig. 5A and solid horizontal line in Fig. 5B). This finding replicates Khuu and Badcock, [31], and demonstrates that the visual system integrates and averages local speed estimates to determine the global object speed. Third, speed averaging was evident when odd-numbered Gabors (moving at a constant speed of 2°/s) were made invisible by CFS. The PSE for this condition was approximately 4°/s and similar to when all elements were visible as in Condition 2. These results demonstrate that the spatial integration of local motion information occurs over visible and invisible elements such that suppressed local estimates continue to be averaged by the visual system to determine global speed.

## General Discussion

The present study comprised of two experiments that examined whether removing local motion signals from awareness affects concurrent conscious determination of global motion. We specifically focussed on the perception of an object's global speed, and determined whether suppressing local elements from awareness modulates the visual system's ability to judge and discriminate global speed. In Experiment 1, we showed that speed discrimination thresholds improved (due to spatial integration) when the number of local elements in the stimulus was increased. However, when the stimulus comprised of both visible and unconscious motion Gabor elements, global speed discrimination reflected integration across both the invisible and visible elements. Here, in general, speed discrimination was superior to baseline conditions in which the number of Gabor elements was physically changed. This result suggests that the integration of local motion can occur across both visible and invisible elements to affect the perception of global speed. In Experiment 2, we report an analogous finding; the visual system is capable of integrating and averaging the speed of local elements that it is not aware of. We show that speed averaging can occur when sourced from both visible and invisible elements in the judgement of global speed. Together, the findings of Experiments 1 and 2 show that the visual system can integrate and combine local motion without visual awareness. This finding agrees with previous studies that have originally documented the unconscious processing of motion by quantifying the MAE, and extends this observation to immediate judgements about an object's motion, including it's speed. In addition, the present study demonstrates that “unseen/invisible” components of a stimulus can influence the motion of the stimulus as whole, such that perception reflects a combination of both visible and invisible motion components. This agrees with previous reports that contextual information (such as spatial orientation) that is suppressed from awareness continues to affect concurrent form perception [25], [27].

The findings of this study agree with Kaunitz et al. [21] who reported MAEs accompanying adaptation to complex spiral motion under CFS. Likewise the present finding of an attenuated integration process is consistent with Kaunitz et al. [21], who also reported an attenuated MAE to spiral motion under CFS conditions. Note that Kaunitz et al. [21] also investigated the role of attention on the extent of the MAE. They observed that focused attention on the adapting stimulus produced greater MAEs compared to when it was unattended. In the present study, observers always observed visible components of the global motion stimulus, which across conditions was varied by selectively suppressing local elements. It is possible that attention to these elements might facilitate the activation of global motion detectors driving them to implicitly integrate local motion information across the visual field. Future research that focuses on clarifying this would be most informative. In particular, whether conscious perception of a stimulus might be a requirement for the integration of unconscious information and elucidating the stimulus conditions under which this occurs.

As the present study is behavioural in nature it is not possible to directly comment on the role of how motion mechanisms might respond to selective attention under CFS conditions. Previous neuroimaging studies have shown that the activation of motion selective areas such as MT is dependent on attention [38]. Future studies employing neural imaging will help elucidate whether and how motion selective areas might respond to motion stimuli under CFS conditions. However, as noted, the dorsal visual pathway subserves the processing of motion [2], [3]. There is evidence that the dorsal pathway remains active when some stimuli are rendered invisible through inter-ocular suppression and that it does not subserve conscious vision [39]. For example, Logothesis and Schall, recorded neural activity to a motion discrimination task under binocular rivalry from the superior temporal sulcus (in monkey and encompassing area MT), which consists of motion selective cells [38]. Here, two different motion patterns were presented separately to the eyes, and single cell recordings were performed under rivalrous viewing conditions. It was observed that while ⅓ of MT cells responded to the perceived dominant motion, ⅓ also responded to the suppressed non-dominant motion, with the last ⅓ showing a mixture of responses. This finding indicates that motion selective cells in higher cortical areas (along the dorsal stream) can effectively and concurrently process both visible and invisible motion signals. The observations made by Logothetis and Schall are certainly consistent with the findings of the present study; we provide behavioural support for the concurrent processing of invisible and visible motion, and that both motion signals effectively contribute to the conscious discrimination and perception of global speed [38].

The present findings contribute to a growing body of literature demonstrating that the processing of motion can occur without conscious perception of the stimulus. Our findings are consistent with those of Kauntiz et al. [23] (see also Chung & Khuu, [22]), who have demonstrated that coherence is an important factor in the processing of global motion without awareness. They showed that global motion patterns of high coherence are likely to break CFS suppression more often than patterns with no coherence. This suggests that the visual system is sensitive to and implicitly processes the motion of the stimulus without awareness. In the present study we show that the perception of global speed operates in a similar manner, and the integration of local motion signals can occur without awareness. Our findings and those of Kauntiz et al. [23] are in agreement with those of Yamada and Kawabe [40] who demonstrated that the perception of ‘high-level’ apparent motion (as the percept is thought be signalled by motion selective areas higher in the processing hierarchy [40]) such as the Line Motion Illusion and transformational apparent motion can occur without being aware of the inducing elements. Importantly, for such motion illusions there is no actual physical object movement (which would otherwise activate low level motion detectors before being integrated by higher cortical areas in a ‘bottom up’ or ‘feed-forward’ manner), but rather motion is *inferred* from briefly presented stationary inducers [41], [42], [43]. It has been shown that area MT is important for the generation of the perception of high-level apparent motion, in particular, the conscious perception of motion might arise from feedback (i.e., ‘top-down’) projections from MT to innervate the retinotopy at V1 [44]. Given the findings of the present study and those of Yamada and Kawabe [40], it is likely that visual awareness might not be requirement for the perception of motion either *inferred* from the spatio-temporal properties of stationary inducers (as with high-level apparent motion) or the movement of actual objects (and reflecting local motion integration).

Though the cortical location underlying conscious visual perception is still a matter of debate and the focus of much research, the findings of the present study are consistent with the view that it must occur after the stage at which local motion is integrated. We show that interocular suppression of local information using CFS does not prevent global motion integration of both visible and invisible elements. Thus, it is possible that motion integration occurs monocularly prior to the stage at which CFS suppresses local elements from awareness. We do note however, that this integration process might be attenuated by the lack of visual awareness of elements. Thus it is possible that while visual awareness is not immediately necessary for the integration of motion, it might improve the detection and discrimination of motion.

While motion integration can occur without awareness, it has not been established whether conscious perception is required for the detection of more complex forms of motion such as biological motion and structure-from-motion [23], [45]. Note that such motion types are examples of motion informing the global form of the stimulus. This process might very well involve inputs from the ventral form pathway [47], which might be reliant on conscious awareness for recognition [22]. Accordingly, such ‘motion forms and shapes’ might be susceptible to binocular suppression, indicating a dependency on visual awareness. In support, Kaunitz et al. [23] have noted that biological motion (which has neural correlates in Superior Temporal Sulcus [46]) does not survive CFS and requires visual awareness for perception. Additionally, Khuu, Alexander, Balcomb and Kim [47] have demonstrated that illusory motion in depth from a moving cast shadow does not arise if the cast shadow is suppressed from awareness using CFS. This study demonstrates that the association between a moving cast shadow and an object in the perception of 3D structure is dependent on visual awareness.

In conclusion, the findings of the present study demonstrate that unconscious motion can contribute to the perception of global motion. In particular the visual system is able to integrate local motion information that is suppressed from awareness to influence the perception and discrimination of global speed. This finding is significant in that it provides further insight into the phenomenology of motion, in particular how visual awareness might modulate the immediate perception and detection of motion for visually guided behaviour.

## Supporting Information

S1 Data **A data file containing psychometric data and speed discrimination thresholds reported in Experiments 1 and 2.**
(XLSX)
